# Prognostic Impact of Active Cigarette Smoking on Mortality in Patients with Acute Venous Thromboembolic Events, Findings from Real World Data

**DOI:** 10.3390/medicina58020295

**Published:** 2022-02-15

**Authors:** Matteo Giorgi-Pierfranceschi, Manuel Monreal, Pierpaolo Di Micco, Iria Francisco, Luis Hernández-Blasco, Olga Madridano, Juan Bosco López-Sáez, Elena Hernando, Jose Meireles, Francesco Dentali, the RIETE Investigators

**Affiliations:** 1 Department of Internal Medicine, Istituti Ospitalieri di Cremona, 26100 Cremona, Italy; 2 Department of Internal Medicine, Hospital Germans Trias i Pujol, Universidad Católica de Murcia, 08016 Barcelona, Spain; mmonreal.germanstrias@gencat.cat; 3 Department of Internal Medicine and Emergency Room, Ospedale Buon Consiglio Fatebenefratelli, 80122 Naples, Italy; pdimicco@libero.it; 4 Department of Internal Medicine, Hospital Universitari de Girona Dr. Josep Trueta, 17007 Gerona, Spain; ifrancisco.girona.ics@gencat.cat; 5 Department of Pneumonology, Hospital General Universitario de Alicante, ISABIAL, UMH, 03014 Alicante, Spain; hernandez_lui@gva.es; 6 Department of Internal Medicine, Hospital Universitario Infanta Sofía, 28042 Madrid, Spain; olgamadridano@hotmail.com; 7 Department of Internal Medicine, Hospital Universitario de Puerto Real, 11006 Cádiz, Spain; juanbosco.lopez@gm.uca.es; 8 Department of Pneumonology, Hospital San Pedro, 26001 Logroño, Spain; ehernando@riojasalud.es; 9 Department of Internal Medicine, Centro Hospitalar de Entre o Douro e Vouga, 4520-195 Santa María da Feira, Portugal; jose.meireles@chedv.min-saude.pt; 10 Department of Medicine and Surgery, Insubria University, 21100 Varese, Italy; fdentali@libero.it

**Keywords:** venous thromboembolism, mortality, cigarette smoking

## Abstract

*Background and Objectives*: The influence of smoking habits on mortality, VTE recurrence, and major bleeding in patients receiving anticoagulant therapy for venous thromboembolism (VTE) has not been consistently evaluated. *Materials and Methods*: We used data from the RIETE (Registro Enfermedad TromboEmbólica) registry to compare mortality, VTE recurrence, and major bleeding risk in smoking versus non-smoking patients with acute VTE. *Results*: 50,881 patients (43,426 non-smoking and 7455 smoking patients) were included. After a median follow-up of 8.8 months, 7110 patients died (fatal PE 292 and fatal bleeding 281), 3243 presented VTE recurrence, and 1579 had major bleeding. At multivariate analysis, smoking behavior was associated with a higher hazard of death, (HR: 1.28; 95% CI: 1.19–1.40). The risk of VTE recurrence was marginally increased in smoking patients compared to non-smoking patients (1.14; 95% CI: 1.02–1.27). Major bleeding did not differ in smoking and non-smoking patients (1.15; 95% CI: 0.96–1.38). The presence of cancer did not appear to influence the association between smoking habits and death (HR: 1.34; 95% CI: 1.22–1.47 in cancer patients and HR: 1.23; 95% CI: 1.04, 1.45 in non-cancer patients, respectively) *Conclusions*: the risk of death after an acute episode of VTE appeared to be higher in smoking than in non-smoking patients and this risk is higher between patients presenting PE at the onset of symptoms.

## 1. Introduction

Venous thromboembolism is one of most frequent cardiovascular diseases associated with death and it is considered as a major health problem because cardiovascular diseases (CVD) are the leading cause of mortality in the general population [1].

Cigarette smoking (CS) may be considered a major risk factor not only for atherothrombosis [e.g., acute coronary syndrome (ACS), ischaemic stroke (IS), and peripheral arterial disease (PAD)] [2,3] but also for pulmonary embolism, according to a meta-analysis of a large cohort of smokers [4]; furthermore, consistent data suggest that CS is associated with a worse survival and event-free survival in these patients [5]. Yet, CS has been also frequently associated as risk factor for the morbidity and mortality of patients affected by venous thromboembolism (VTE), although data in the literature are less compelling [6,7]. In particular, data about the prognostic influence of CS in patients after an episode of VTE are lacking. Furthermore, the influence of smoking habits during anticoagulation for a VTE is really complex to understand and its effect on mortality, VTE recurrence, and major bleeding has not been consistently evaluated after a VTE. In particular, in the literature, smoking habits have been associated with an increased risk for VTE, but there is scarce evidence on the risk for VTE recurrences in patients who keep smoking after VTE. We hypothesized that a big increase in the risk for recurrences (if demonstrated) should be accompanied by some modifications in the intensity and/or the duration of anticoagulation. Thus, the aim of the current study was to compare mortality rates and risk of recurrences of VTE during follow-up according to the use of smoking at the time of the indexed VTE event, using data from the RIETE (Registro Enfermedad TromboEmbólica) registry.

## 2. Patients and Methods

The RIETE (Registro Informatizado Enfermedad TromboEmbólica) registry is an ongoing, multicenter, observational registry of consecutive patients with objectively confirmed, acute VTE (ClinicalTrials.gov identifier: NCT02832245). The rationale and methodology of RIETE have been published elsewhere. RIETE is an ongoing, multicenter registry of consecutive patients with objectively confirmed acute VTE (ClinicalTrials.gov identifier: NCT02832245). It started in Spain in 2001 and, after 6 years, it expanded to other countries. Currently, RIETE includes 254 collaborating centers in 27 countries.

### 2.1. Patients

Consecutive patients with symptomatic acute deep vein thrombosis (DVT) confirmed by compression ultrasonography, or pulmonary embolism (PE), confirmed by helical CT-scan, and ventilation-perfusion lung scintigraphy or angiography, were enrolled in the RIETE. Patients were excluded if they were currently participating in a therapeutic blinded trial with a blinded therapy. All patients or their relatives provided written or oral informed consent for participation in the registry, in accordance with the local ethics committee requirements.

### 2.2. Study Design

For the current study, only patients with information on the smoking use at the index VTE were considered. As information on current smoking habits were included in the RIETE registry in February 2009, patients enrolled in the registry before February 2009 were not included in this analysis. In secondary analyses, patients with DVT (without a concomitant PE), patients with PE (with or without a concomitant DVT), and patients without active cancer (defined as newly diagnosed cancer or cancer that is being treated (i.e., surgery, chemotherapy, radiotherapy, support therapy, or combined treatment) were separately analyzed.

Consecutive patients with symptomatic acute deep vein thrombosis (DVT) or pulmonary embolism (PE), confirmed by objective tests (compression ultrasonography or contrast venography for DVT; helical CT-scan, ventilation-perfusion lung scintigraphy or angiography for PE), were enrolled in the RIETE. RIETE’s rationale and methodology has recently been published [7,8]. Patients were excluded if they were currently participating in a therapeutic clinical trial with a blinded therapy. All patients (or their relatives) provided written or oral informed consent for participation in the registry, in accordance with local ethics committee requirements.

For the current study, we selected patients with acute VTE comparing active smokers to non-smokers. Patients enrolled in the RIETE registry before February 2009 could not be included, as data on smoking habits were not collected. In secondary analyses, patients with DVT (without a concomitant PE), patients with PE (with or without a concomitant DVT), and patients without active cancer (defined as newly diagnosed cancer or cancer that is being treated [surgery, chemotherapy, radiotherapy, support therapy, or combined treatments]) were separately analyzed.

### 2.3. Outcomes

The major outcome was the occurrence of death during follow-up after an acute episode of VTE. Secondary outcomes were VTE recurrence and major bleeding. In order to evaluate VTE recurrences, a clinical and instrumental follow up was required for at least 90 days since first documented VTE. Fatal PE, in the absence of autopsy, was defined as any death appearing within 10 days of a symptomatic objectively confirmed PE event, in the absence of any alternative cause of death. Major bleeding was defined as retroperitoneal, spinal, intracranial, or fatal bleeding or as any overt bleeding requiring transfusion of two or more units of blood. Fatal bleeding was defined as any death occurring within 10 days of a major bleeding episode, in the absence of an alternative cause of death.

Finally, exploratory analyses of different causes of death were evaluated (e.g., recurrent PE, bleeding, sudden death, respiratory insufficiency, infection, or disseminated cancer).

### 2.4. Baseline Variables

The following parameters were recorded when the qualifying episode of VTE was diagnosed: the patient’s baseline characteristics; clinical status including chronic heart or lung disease, recent major bleeding, anemia, thrombocytopenia, or renal insufficiency; site of cancer, presence or absence of metastases and type of oncologic therapy; additional risk factors for VTE; the treatment received upon VTE diagnosis (drug, dose, date of start, and date of discontinuation for each drug); concomitant drugs and the outcomes during the course of anticoagulant therapy. Immobilized patients were defined as non-surgical patients who had been immobilized (i.e., total bed rest with or without bathroom privileges) for ≥4 days in the 2-months prior to the index VTE. Surgical patients were defined as those who had undergone an operation in the 2 months prior to VTE. Anemia was defined as hemoglobin levels <13 g/dL for men and <12 g/dL for women. Creatinine clearance (CrCl) levels at baseline were measured according to the Cockcroft and Gault formula.

### 2.5. Treatment and Follow-Up

Patients were managed according to the clinical practice of each participating hospital (i.e., there was no standardization of therapy). The type, dose, and duration of anticoagulant therapy were recorded. After VTE diagnosis, patients were followed up with in the outpatient clinic for at least 3 months. During each visit, any signs or symptoms suggesting VTE recurrences or bleeding complications were noted. Each episode of clinically suspected recurrent DVT or PE was investigated by repeat compression ultrasonography, lung scanning, helical-CT scan, or pulmonary angiography, as appropriate.

### 2.6. Statistical Analysis

All data are reported as mean ± standard deviations. Specific variables were looked for, selecting those more commonly associated to our goals between those available to the registry in an exploratory way. Differences in mean values between groups were assessed using the Student’s *t*-test analysis. Categorical variables were compared using the chi-square test or fisher exact test, when appropriate. Associations between smoke and mortality were assessed using the multivariate Cox proportional hazards regression model, in which age, gender, risk factors for VTE, prior VTE, recent major bleeding, creatinine clearance levels, anemia, abnormal platelet count, type, and duration of treatment and clinical presentation of VTE at baseline (PE vs. DVT alone) were adjusted as confounding factors. Hazard ratios and corresponding 95% confidence intervals were calculated. Normality was verified using the Shapiro-Wilks test and graphically using the Q-Q plot. Given the obvious association between active cancer and smoke, we separately analyzed patients without active cancer. Statistical analyses were conducted with SAS software 9.4 (SAS Institute Inc., Cary, NC, USA).

## 3. Results

### 3.1. Clinical Characteristics

From March 2001 to June 2019, 79,301 patients with acute VTE were enrolled in the RIETE registry. Of these, 43,426 patients were non-smokers and 7455 patients were active smokers, while for the remaining patients (28,420 patients; 35.8% of the whole sample) data on smoking attitude was not recorded (because patients were included before February 2009 or because this information is lacking). Thus, 50,881 patients were included in this analysis. Smoking patients were more likely men, were younger, and were more likely to have chronic lung disease compared to non-smoking patients. Conversely, they were less likely to have chronic heart failure, arterial hypertension, diabetes, anemia, or abnormal platelet count at baseline, Table 1. Overall, 1405 smoking patients (18.9%) and 10,319 non-smoking patients (23.8%) had active cancer. Median and total follow up were 8.8 months (interquartile range 4.1–20.2 months) and 68,427.93 patient years, respectively.

Information on acute and long-term antithrombotic therapy was available for 50,728 patients (99.7% of the whole sample). Duration of anticoagulant therapy was similar in smoking and non-smoking patients; some small but significant differences were found in the initial and long-term anticoagulant therapy between these two groups, Table 2. Cancer patients were more likely to receive low-molecular-weight heparin (LMWH) for long-term treatment but there were no significant differences in smoking and non-smoking cancer patients (data not shown).

### 3.2. Clinical Outcomes

During follow-up, 7110 patients died (10.4 per 100 patients–year; fatal PE 292; fatal bleeding 281), 3243 presented VTE recurrence and 1579 had major bleeding. As it could be expected, patients with cancer had higher rates of VTE recurrence, major bleeding, and death than those without cancer, Table 3. Patients presenting with PE had a lower rate of VTE recurrence and a higher rate of major bleeding compared to patients presenting with DVT. Median time for VTE recurrence in smokers was 1.9 years. Mortality was similar in these two groups.

### 3.3. Multivariate Analysis

At multivariate analysis analysis, smoking patients appeared to have a slightly higher VTE recurrence rate (5.6 vs. 5.0; HR: 1.1, 95% CI: 1.0, 1.2) and a lower rate of death (8.0 vs. 10.8) and of major bleeding (1.7 vs. 2.5). Results obtained in PE and in DVT patients as well as in patients with and without cancer, were summarized in Table 3.

At multivariate analysis, smoking behavior was associated with a higher hazard of death, (HR: 1.28; 95% CI: 1.19–1.40), Table 4. Risk of VTE recurrence was marginally increased in smoking patients compared to non-smoking patients (1.14; 95% CI: 1.02–1.27). Major bleeding did not differ in smoking and non-smoking patients (1.15; 95% CI: 0.96–1.38). Other factors associated with a higher hazard of death were the following: cancer (HR: 5.17; 95% CI: 4.90–5.46), immobility (HR: 1.70; 95% CI: 1.61–1.80), chronic heart failure (HR: 1.50; 95% CI: 1.39–1.62), chronic lung disease (HR: 1.15; 95% CI: 1.07–1.23) and diabetes (HR: 1.18; 95% CI: 1.11–1.26).

In both the subgroup of patients presenting with PE and DVT, the association between smoking behavior and death was maintained (1.35; 95% CI: 1.21–1.50; 1.14; 95% CI: 1.00–1.31, respectively).

The presence of cancer did not appear to influence the association between smoking habits and death (HR: 1.34; 95% CI: 1.22–1.47 in cancer patients and HR: 1.23; 95% CI: 1.04, 1.45 in non-cancer patients, respectively).

Cardiovascular causes of death (recurrent PE, myocardial infarction, and critical limb ischemia) appeared more common in smoking patients but this risk did not appear to be significant. Appendix A.

## 4. Discussion

CS is associated with a worse survival and event-free survival in patients presenting with arterial cardiovascular events (namely ACS, stroke, and PAD) [4]. To date, to our knowledge, few studies have addressed the prognostic role of CS in patients presenting with an acute episode of VTE smoke [9,10]. As the risk of VTE recurrences was only marginally increased in smoking patients compared to non-smoking patients (HR: 1.14), no treatment modifications seem to be justified.

Our findings, obtained from a large cohort of patients with VTE and after adjusting for a number of potentially confounding variables, reveal that smoking patients presenting with an acute episode of VTE are at higher risk of death than non-smoking patients. These results are consistently found in patients with or without cancer, in patients presenting with DVT and in patients presenting with PE with a stronger association between CS and mortality in the latter group.

A higher risk of death in smoking patients was uniformly found for most cardiovascular causes of death (recurrent PE, myocardial infarction, and critical limb ischemia), although it did not reach statistically significant differences, most likely because of the sample size. Risk of VTE recurrence was marginally increased in smoking patients compared to non-smoking patients (1.14; 95% CI: 1.02–1.27), while major bleeding did not significantly differ in these two groups of patients (1.15; 95% CI: 0.96–1.38).

Previous studies with conflicting results have assessed the association between VTE and smoke [5,6,11,12]. In an old systematic review and meta-analysis of the literature by Ageno et al., smoke did not appear to be associated with a first episode of VTE [5]. Conversely, Cheng et al. [7] and Mahmoodi M et al. [4] reported a slightly increased risk of VTE in smoking patients. In another work that used data from the Atherosclerosis Risk in Communities study (ARIC), the association between cardiovascular risk factors and incident VTE was evaluated in a cohort of 15,340 participants who had no history of VTE and/or anticoagulant use at enrolment [12]. A mean follow-up time of 15.5 years (237,375 person-years) and smoking status, after adjustment for demographic variables and BMI, resulted to be associated with an increased risk of VTE (hazard ratio 1.44; 95% CI: 1.12–1.86)

In the Tromsø Study, information on smoking habits was assessed by self-administered questionnaires in 24.576 subjects aged 25–96 years [11]. After a mean follow-up of 12.5 years, heavy smoking was found to be a risk factor for provoked VTE in the analyses with VTE events as the only outcome, but the lack of association between smoking and VTE risk in the analyses censored at the occurrence of cancer [13] or myocardial infarction, suggests that smoking-attributable diseases or other predisposing factors are essential to convey a risk of VTE.

A meta-analysis of 20 studies demonstrated a 36% reduction in the crude relative risk of mortality for patients with coronary heart disease who quit smoking compared with those who continued smoking [14]. Smoking cessation also leads to an exponential reduction in acute cardiovascular events, particularly in the first year after quitting [15].

Evidence on the role of CS as a potential risk factor for VTE recurrence and as a prognostic factor in patients with an acute episode of VTE is even more limited, as no large high-quality prospective study has evaluated this aspect.

CS has many potential effects on hemostatic balance [16]: through different mechanisms, it may reduce nitric oxide bioavailability potentially affecting inflammation, leukocyte adhesion, and platelet activation [17,18]; CS may cause an imbalance in endothelial cell (EC) derived antithrombotic and fibrinolytic factors (i.e., an increase in circulating tissue factor (TF) activity and a significant decrease in EC-derived tissue factor pathway inhibitor 1 secretion) [16,17,18]; platelet α-granule constituents, such as platelet factor-4, β-thromboglobulin, and the platelet activating factor, are increased in smokers’ plasma, suggesting increased platelet activation [19,20,21]. These and other data [22] suggest the existence of a pro-thrombotic state in these patients leading to an increased risk of arterial and venous thromboembolic events.

Conflicting results depending on the current smoking status of the patients, with some authors reporting an increased risk of VTE among current smokers only and also among heavy smokers only [23], as far as for the risk of mortality in same subgroups of smokers regarding lung cancer or cardiovascular diseases [24,25]. Yet, in our population we found, interestingly, a low bleeding rate (minor and major events) associated with a slight increase of VTE recurrences.

Although mortality risk appeared only modestly increased in smoking patients, such an increase becomes relevant and numerically meaningful considering how mortality risk is already high at baseline and how represented smokers are in this population.

Thus, considering the total number of smoking patients affected by VTE, lowering said mortality risk by promoting the quitting of smoking habits may result in a relevant intervention and creates another valuable reason to support the effort of many countries that have already started tobacco control programs that are showing considerable success.

Furthermore, based on our results on a large population of a real world study, we could underline that there is a linear correlation starting from the pathophysiological effects of the prothrombotic effects of smoking on haemostatic balance and clinical events regarding arterial and venous thromboses to the clinical outcomes that may stress the clinical concept of increased rate of recurrences in smokers vs. non-smokers as far as the reduced rate of bleeding complication. Therefore, we should probably speculate that smokers with recent VTE could take advantage of a stronger anticoagulant treatment regarding intensity of anticoagulant treatment or its duration but confirmation is needed by randomized clinical trials.

Our study has some limitations. First, our results should be treated with caution considering how observational studies could be limited by inferring causality. Confounding is a potential limitation of the cohort design, due to the absence of random allocation. However, in our study, we were able to adjust our results for many prospectively collected variables minimizing this risk. Furthermore, in the RIETE registry, information on smoking habits is collected only at time of enrolment, while the smoking behavior could change over time, making it impossible to know whether a smoking habit had been stopped or taken up during follow-up and thus potentially creating a misclassification in our analysis. Also, unfortunately, due to limitations in data collection, we could only classify patients as smokers or non-smokers without further information, and thus have no insights on the potential role of dose and duration of smoking. Finally, smoking habits were self-reported, making it possible for such information to be insincere or inaccurate. However, self-reporting of smoking habits has been proven to have high validity and reliability [26].

In conclusion, risk of death after an acute episode of VTE appeared to be higher in smoking than in non-smoking patients. This seems to be especially true in patients presenting with PE. Other studies should confirm our preliminary findings and should assess if active campaigns to quit smoking may be useful in reducing mortality rates among these patients.

## Figures and Tables

**Table 1:** Clinical characteristics of the patients according to smoking behavior at baseline.

	Total	Non Smoker	Smoker	*p*-Value
Patients, N	50,881	43,426	7455	
**Clinical characteristics**				
Male gender	24,794 (48.7%)	19,726 (45.4%)	5068 (68.0%)	<0.0001
Age (years ± SD)	65.1 ± 17.7	67.1 ± 17.1	53.0 ± 15.8	<0.0001
Body weight (kg ± SD)	76.4 ± 16.6	75.9 ± 16.2	79.6 ± 18.4	<0.0001
**VTE characteristics**				
Symptomatic PE at baseline	28,487 (56.0%)	24,451 (56.3%)	4036 (54.1%)	0.01
**Risk factors for VTE**				
Surgery	5565 (10.9%)	4858 (11.2%)	707 (9.5%)	<0.0001
Immobility	11,111 (21.8%)	9788 (22.5%)	1323 (17.8%)	<0.0001
Estrogen intake	3217 (6.5%)	2492 (5.7%)	725 (9.9%)	<0.0001
Pregnancy/puerperium	641 (2.5%)	573 (2.4%)	68 (2.8%)	0.19
Cancer	11,724 (23.0%)	10,319 (23.8%)	1405 (18.9%)	<0.0001
None of the above (unprovoked)	10,611 (20.9%)	9793 (22.6%)	818 (11.0%)	<0.0001
Prior VTE	7682 (15.1)	6577 (15.1%)	1105 (14.8%)	0.47
**Underlying diseases**				
Chronic lung disease	6203 (12.2%)	5130 (11.8%)	1073 (14.4%)	<0.0001
Chronic heart failure	3623 (7.1%)	3307 (7.6%)	316 (4.2%)	<0.0001
Arterial hypertension	23,851 (47.1%)	21,657 (50.0%)	2194 (29.8%)	<0.0001
Diabetes	7696 (15.2%)	6877 (15.9%)	819 (11.1%)	<0.0001
**Laboratory tests**				
Renal function, N	43499	37154	6345	
CrCL levels (mean mL/min ± SD)	87.8 ± 46.1	83.3 ± 46.1	113.8 ± 51.6	<0.0001
CrCL levels >60 mL min	29,943 (68.8%)	24,403 (65.7%)	5540 (87.3%)	<0.0001
CrCL levels 30–60 mL min	10,900 (25.1%)	10,223 (27.5%)	677 (10.7%)	<0.0001
CrCL levels <30 mL min	2656 (6.1%)	2528 (6.8%)	128 (2.0%)	<0.0001
Anaemia	17,264 (34.0%)	15,353 (35.4%)	1911 (25.7%)	<0.0001
Abnormal platelet count	9900 (19.5%)	8575 (19.8%)	1325 (17.8%)	<0.0001

Percentage calculated on the number of patients with data available. Legend to Table 1: HR, hazard ratio; CI, confidence intervals; PE, pulmonary embolism; CrCl, creatinine clearance; DVT, deep vein thrombosis. Data of continuous variables were expressed as mean ± SD.

**Table 2:** Treatment strategies according to smoking behavior at baseline and the presence or absence of cancer.

	Total	All			With Cancer			Without Cancer		
		Non Smoker	Smoker	*p*-Value	Non Smoker	Smoker	*p*-Value	Non Smoker	Smoker	*p*-Value
Patients, N	50,728	43,294	7434		10,268	1246		33,026	6039	
**Duration of therapy**										
Mean months (SD)	10.2 (13.8)	10.2 (13.9)	10.0 (13.1)	0.27	8.5 (12,1)	7.4 (10.9)	0.001	10.8 (14.4)	10.7 (13.5)	0.55
Median months (IQR)	6.1 (3.4; 11.7)	6.1 (3.4; 11.7)	6.2 (3.5; 11.6)		5.0 (3.0; 9.4)	4.3 (2.6; 8.2)		6.2 (3.5; 12.0)	6.4 (3.7; 12.0)	
**Initial therapy**										
LMWH	43,997 (86.7%)	37,654 (87%)	6343 (85.3)	0.0001	9315 (90.7%)	1246 (89.3%)	0.09	28,339 (85.8%)	5097 (84.4%)	0.04
UFH	2544 (5.0%)	2124 (4.9%)	420 (5.7%)	0.01	497 (4.8%)	90 (6.5%)	0.01	1626 (4.9%)	330 (5.5%)	0.08
Penta-saccaride	1376 (2.7%)	1153 (2.7%)	223 (3.0%)	0.10	178 (1.7%)	29 (2.1%)	0.36	975 (3.0)	194 (3.2)	0.28
DOACs	1601 (3.2%)	1366 (3.2%)	235 (3.2%)	0.98	137 (1.3%)	12 (0.9%)	0.14	1229 (3.7%)	223 (3.7)	0.91
Thrombolytics	771 (1.5%)	622 (1.4%)	149 (2.0%)	0.0002	74 (0.7%)	10 (0.7%)	0.99	548 (1.7)	139 (2.3)	0.001
Others	125 (0.2%)	115 (0.3%)	10 (0.1%)	0.04	30 (0.3%)	4 (0.3%)	0.97	85 (0.3)	6 (0.1)	0.02
Biosimilar of Enoxaparin	15 (0.03%)	13 (0.03%)	2 (0.03%)	0.88	3 (0.3%)	0 (0.0%)	0.52	10 (0.3)	2 (0.03)	0.91
**Patients, N**	**49,633**	**42,307**	**7326**		**9789**	**1327**		**32,518**	**5999**	
**Long-term therapy**										
LMWH	16,552 (33.3%)	14,368 (34.0%)	2184 (29.8)	<0.0001	6884 (70.3%)	957 (72.1)	0.18	7484 (23.0%)	1227 (20.5)	<0.0001
UFH	208 (0.4%)	173 (0.4)	35 (0.5)	0.40	83 (0.9)	22 (1.7)	0.004	90 (0.3)	13 (0.2)	0.41
DOACS	6675 (13.4%)	5694 (13.5)	981 (13.4)	0.87	648 (6.6)	80 (6.0)	0.41	5046 (15.5)	901 (15.0)	0.33
VKA	31,596 (63.7%)	26,659 (63.0)	4937 (67.4)	<0.0001	3292 (33.6)	407 (30.7)	0.03	23,367 (71.9)	4530 (75.5)	<0.0001
Penta-saccaride	588 (1.2%)	500 (1.2)	88 (1.2)	0.89	173 (1.8)	24 (1.8)	0.91	327 (1.0)	64 (1.1)	0.66
Others	279 (0.6%)	239 (0.6)	40 (0.6)	0.84	46 (0.5)	10 (0.8)	0.17	193 (0.6)	30 (0.5)	0.38
Biosimilar of Enoxaparin	8 (0.02%)	7 (0.02)	1 (0.01)	0.86	5 (0.05)	0 (0.0)	0.41	2 (0.01)	1 (0.02)	0.40

Legend to Table 2: HR, hazard ratio; CI, confidence intervals; PE, pulmonary embolism; CrCl, creatinine clearance; DVT, deep vein thrombosis.

**Table 3:** Clinical outcomes during anticoagulant therapy in the whole sample and according to smoking behavior.

	All		Non Smoker			Smoker		
	N	Incidence (per 100 PY)	N	Incidence (per 100 PY)	HR (95% CI)	N	Incidence (per 100 PY)	HR (95% CI)
All Patients, N	50,881		43,426			7455		
Recurrent VTE	3243	5.1 (4.9; 5.2)	2717	5.0 (4.8; 5.2)	1.0 (ref.)	526	5.6 (5.1; 6.0)	1.1 (1.0; 1.2)
Major bleeding	1579	2.4 (2.2; 2.5)	1412	2.5 (2.3; 2.6)	1.0 (ref.)	167	1.7 (1.4; 2.0)	0.68 (0.58; 0.80)
Death	7110	10.4 (10.2; 10.6)	6297	10.8 (10.5; 11.1)	1.0 (ref.)	813	8.0 (7.5; 8.6)	0.74 (0.69; 0.80)
Fatal PE	292	0.4 (0.38; 0.48)	254	0.4 (0.39; 0.49)	1.0 (ref.)	38	0.38 (0.27; 0.52)	0.9 (0.62; 1.22)
Fatal bleeding	281	0.4 (0.37; 0.46)	256	0.4 (0.39; 0.50)	1.0 (ref.)	25	0.2 (0.17; 0.37)	0.56 (0.37; 0.85)
**Patients with cancer, N**	**11,724**		**10,319**			**1405**		
Recurrent VTE	979	8.1 (7.6; 8.6)	832	7.7 (7.2; 8.2)	1.0 (ref.)	147	11.5 (9.8; 13.5)	1.46 (1.22; 1.73)
Major bleeding	601	4.7 (4.3; 5.1)	518	4.5 (4.1; 4.9)	1.0 (ref.)	83	6.1 (4.9; 7.6)	1.25 (0.99; 1.58)
Death	4434	33.6 (32.6; 34.6)	3808	32.3 (31.3; 33.3)	1.0 (ref.)	626	44.7 (41.3; 48.3)	1.30 (1.20; 1.42)
Fatal PE	130	1.0 (0.82; 1.17)	107	0.9 (0.8; 1.1)	1.0 (ref.)	23	1.6 (1.1; 2.5)	1.60 (1.02; 2.52)
Fatal bleeding	126	0.95 (0.80; 1.14)	110	0.9 (0.8; 1.1)	1.0 (ref.)	16	1.1 (0.7; 1.9)	1.14 (0.67; 1.92)
**Patients without cancer, N**	**39,157**		**33,107**			**6050**		
Recurrent VTE	2264	4.3 (4.2; 4.5)	1885	4.3 (4.1; 4.5)	1.0 (ref.)	379	4.6 (4.2; 5.1)	1.08 (0.96; 1.21)
Major bleeding	978	1.8 (1.7; 1.9)	894	2.0 (1.8; 2.1)	1.0 (ref.)	84	1.0 (0.8; 1.2)	0.50 (0.40; 0.63)
Death	2676	4.8 (4.7; 5.0)	2489	5.4 (5.1; 5.6)	1.0 (ref.)	187	2.1 (1.9; 2.5)	0.40 (0.35; 0.47)
Fatal PE	162	0.3 (0.25; 0.34)	147	0.3 (0.2; 0.4)	1.0 (ref.)	15	0.2 (0.1; 0.3)	0.56 (0.33; 0.95)
Fatal bleeding	155	0.3 (0.24; 0.33)	146	0.3 (0.3; 0.4)	1.0 (ref.)	9	0.1 (0.05; 0.2)	0.33 (0.17; 0.65)
**Patients with EP (with or without DVT), N**	**28,487**		**24,451**			**4036**		
Recurrent VTE	1585	4.3 (4.1; 4.5)	1343	4.2 (4.0; 4.5)	1.0 (ref.)	242	4.6 (4.1; 5.6)	1.10 (0.96; 1.26)
Major bleeding	996	2.6 (2.4; 2.8)	886	2.7 (2.5; 2.9)	1.0 (ref.)	110	2.0 (1.7; 2.4)	0.75 (0.61; 0.91)
Death	4285	11.0 (10.7; 11.3)	3788	11.3 (11.0; 11.7)	1.0 (ref.)	497	9.0 (8.2; 9.8)	0.79 (0.72; 0.87)
Fatal PE	265	0.7 (0.6; 0.8)	234	0.7 (0.6; 0.9)	1.0 (ref.)	31	0.6 (0.4; 0.8)	0.80 (0.55; 1.16)
Fatal bleeding	193	0.49 (0.43; 0.57)	174	0.5 (0.4; 0.6)	1.0 (ref.)	19	0.3 (0.2; 0.5)	0.66 (0.41; 1.06)
**Patients with DVT (no EP), N**	**22,394**		**18,975**			**3419**		
Recurrent VTE	1658	6.1 (5.8; 6.4)	1374	6.0 (5.7; 6.3)	1.0 (ref.)	284	6.7 (6.0; 7.5)	1.12 (0.98; 1.28)
Major bleeding	583	2.0 (1.9; 2.2)	526	2.2 (2.0; 2.3)	1.0 (ref.)	57	1.3 (1.0; 1.6)	0.59 (0.45; 0.77)
Death	2825	9.6 (9.3; 10.0)	2509	10.1 (9.7; 10.5)	1.0 (ref.)	316	6.9 (6.2; 7.7)	0.68 (0.61; 0.77)
Fatal PE	21	0.09 (0.06; 0.13)	20	0.08 (0.05; 0.1)	1.0 (ref.)	7	0.2 (0.07; 0.3)	1.91 (0.81; 4.51)
Fatal bleeding	88	0.3 (0.2; 0.4)	82	0.3 (0.3; 0.4)	1.0 (ref.)	6	0.1 (0.06; 0.3)	0.40 (0.17; 0.91)
**Patients with EP (with or without DVT) without cancer, N**	**22,111**		**18,837**			**3274**		
Recurrent VTE	1143	3.8 (3.6; 4.0)	968	3.8 (3.5; 4.0)	1.0 (ref.)	175	3.8 (3.3; 4.5)	1.03 (0.87; 1.21)
Major bleeding	648	2.1 (1.9; 2.2)	590	2.2 (2.1; 2.4)	1.0 (ref.)	58	1.2 (0.9; 1.6)	0.55 (0.42; 0.72)
Death	1803	5.7 (5.4; 5.9)	1674	6.2 (5.9; 6.5)	1.0 (ref.)	129	2.7 (2.3; 3.2)	0.44 (0.36; 0.52)
Fatal PE	152	0.5 (0.4; 0.6)	140	0.5 (0.4; 0.6)	1.0 (ref.)	12	0.2 (0.1; 0.4)	0.49 (0.27; 0.89)
Fatal bleeding	119	0.4 (0.3; 0.4)	113	0.4 (0.3; 0.5)	1.0 (ref.)	6	0.1 (0.06; 0.3)	0.30 (0.13; 0.68)
**Patients with DVT (no EP) without cancer, N**	**17,046**		**14,270**			**2776**		
Recurrent VTE	1121	5.1 (4.9; 5.4)	917	5.0 (4.7; 5.4)	1.0 (ref.)	204	5.6 (4.9; 6.4)	1.12 (0.96; 1.30)
Major bleeding	330	1.4 (1.3; 1.6)	304	1.6 (1.4; 1.8)	1.0 (ref.)	26	0.7 (0.5; 1.0)	0.43 (0.29; 0.64)
Death	873	3.7 (3.5; 4.0)	815	4.2 (3.9; 4.5)	1.0 (ref.)	58	1.5 (1.1; 1.9)	0.36 (0.27; 0.47)
Fatal PE	10	0.04 (0.02; 0.08)	7	0.04 (0.02; 0.08)	1.0 (ref.)	3	0.08 (0.02; 0.24)	2.16 (0.56; 8.35)
Fatal bleeding	36	0.15 (0.11; 0.21)	33	0.2 (0.1; 0.2)	1.0 (ref.)	3	0.08 (0.02; 0.24)	0.46 (0.14; 1.49)
**Patients with EP (with or without DVT) with cancer, N**	**6376**		**5614**			**762**		
Recurrent VTE	442	6.6 (6.0; 7.3)	375	6.3 (5.7; 6.9)	1.0 (ref.)	67	10.0 (7.9; 12.7)	1.54 (1.19; 2.00)
Major bleeding	348	5.0 (4.5; 5.6)	296	4.8 (4.3; 5.4)	1.0 (ref.)	52	7.5 (5.7; 9.8)	1.41 (1.05; 1.89)
Death	2482	34.7 (33.4; 36.1)	2114	32.9 (31.5; 34.3)	1.0 (ref.)	368	51.0 (46.0; 56.5)	1.43 (1.28; 1.59)
Fatal PE	113	1.6 (1.3; 1.9)	94	1.5 (1.2; 1.8)	1.0 (ref.)	19	2.6 (1.7; 4.1)	1.52 (0.93; 2.48)
Fatal bleeding	74	1.03 (0.82; 1.30)	61	0.9 (0.7; 1.2)	1.0 (ref.)	13	1.8 (1.0; 3.1)	1.73 (0.95; 3.15)
**Patients with DVT (no EP) with cancer, N**	**5348**		**4705**			**643**		
Recurrent VTE	537	9.8 (9.0; 10.7)	457	9.4 (8.6; 10.3)	1.0 (ref.)	80	13.1 (10.5; 16.3)	1.37 (1.08; 1.74)
Major bleeding	253	4.3 (3.8; 4.8)	222	4.2 (3.7; 4.8)	1.0 (ref.)	31	4.7 (3.3; 6.7)	1.06 (0.73; 1.54)
Death	1952	32.3 (30.8; 33.7)	1694	32.6 (30.1; 33.1)	1.0 (ref.)	258	37.9 (33.6; 42.9)	1.15 (1.01; 1.32)
Fatal PE	17	0.3 (0.17; 0.45)	13	0.2 (0.1; 0.4)	1.0 (ref.)	4	0.6 (0.2; 1.6)	2.28 (0.74; 6.98)
Fatal bleeding	52	0.9 (0.7; 1.13)	49	0.9 (0.7; 1.2)	1.0 (ref.)	3	0.4 (0.1; 1.4)	0.46 (0.14; 1.47)

Legend to Table 3: HR, hazard ratio; CI, confidence intervals; PE, pulmonary embolism; CrCl, creatinine clearance; DVT, deep vein thrombosis; Ref., reference.

**Table 4:** Multivariate analyses for all-cause mortality, VTE recurrences, and major bleeding in patients with or without cancer. Results are expressed as hazard ratio and 95% confidence intervals.

	All Sample					
	VTE Recurrence		Major Bleeding		Death	
	HR	95% CI	HR	95% CI	HR	95% CI
**All Patients**						
SMOKE						
No	1.0	Ref	1.0	ref	1.0	Ref
Yes	1.14	1.02; 1.27	1.15	0.96; 1.38	1.28	1.19; 1.40
Sex						
Male	1.0	Ref	1.0	ref	1.0	Ref
Female	0.89	0.83; 0.97	1.12	1.00; 1.26	0.91	0.86; 0.96
Therapy duration (months)	0.93	0.92; 0.93	0.92	0.91; 0.93	0.96	0.96; 0.97
Age	1.00	0.99; 1.01	1.02	1.01; 1.02	1.03	1.02; 1.03
Symptomatic PE	0.93	0.86; 1.00	1.54	1.38; 1.72	1.31	1.25; 1.38
Surgery	0.55	0.48; 0.64	0.89	0.75; 10.6	0.58	0.53; 0.64
Immobility	0.90	0.82; 0.99	1.21	1.07; 1.37	1.70	1.61; 1.80
Estrogen intake	0.57	0.47; 0.70	0.80	0.61; 1.06	0.50	0.43; 0.57
Cancer	1.76	1.61; 1.92	1.80	1.60; 2.02	5.17	4.90; 5.46
Chronic lung disease	1.05	0.94; 1.19	1.16	1.00; 1.34	1.15	1.07; 1.23
Chronic heart failure	1.06	0.90; 1.26	1.18	1.00; 1.40	1.50	1.39; 1.62
Arterial hypertension	1.07	0.98; 1.17	1.09	0.97; 1.23	0.78	0.74; 0.83
Diabetes	0.97	0.87; 1.09	0.93	0.80; 1.07	1.18	1.11; 1.26
CrCL levels						
>60 mL min	1.0	Ref	1.0	ref	1.0	Ref
30–60 mL min	1.12	1.01; 1.24	1.32	1.16; 1.52	1.30	1.22; 1.39
<30 mL min	1.06	0.89; 1.27	1.90	1.58; 2.29	2.14	1.97; 2.32
Anaemia	0.91	0.83; 0.99	1.82	1.62; 2.04	1.65	1.56; 1.74
Abnormal platelet count	1.07	0.98; 1.18	1.39	1.23; 1.57	1.39	1.31; 1.47
**Patients without cancer**						
SMOKE						
No	1.0	Ref	1.0	ref	1.0	Ref
Yes	1.08	0.95; 1.23	1.05	0.82; 1.35	1.23	1.04; 1.45
Sex						
Male	1.0	Ref	1.0	ref	1.0	Ref
Female	0.87	0.79; 0.96	1.12	0.97; 1.30	0.85	0.78; 0.92
Therapy duration (months)	0.93	0.92; 0.94	0.93	0.92; 0.94	0.92	0.91; 0.93
Age	1.00	0.99; 1.01	1.02	1.01; 1.03	1.06	1.05; 1.07
Symptomatic PE	0.91	0.83; 1.00	1.54	1.33; 1.78	1.60	1.46; 1.74
Surgery	0.59	0.49; 0.71	0.85	0.67; 1.09	0.78	0.66; 0.92
Immobility	0.91	0.81; 1.02	1.16	1.00; 1.35	1.96	1.80; 2.13
Estrogen intake	0.47	0.35; 0.62	0.78	0.49; 1.26	1.16	0.81; 1.67
Chronic lung disease	1.04	0.90; 1.20	1.12	0.93; 1.35	1.39	1.26; 1.54
Chronic heart failure	1.06	0.87; 1.29	1.29	1.05; 1.57	1.74	1.58; 1.93
Arterial hypertension	1.06	0.96; 1.18	1.13	0.97; 1.33	0.80	0.73; 0.87
Diabetes	0.91	0.79; 1.05	0.92	0.77; 1.10	1.40	1.27; 1.54
CrCL levels						
>60 mL min	1.0	Ref	1.0	ref	1.0	Ref
30–60 mL min	1.19	1.05; 1.35	1.48	1.24; 1.76	1.47	1.32; 1.63
<30 mL min	1.19	0.96; 1.47	2.17	1.73; 2.73	2.46	2.16; 2.80
Anaemia	0.87	0.77; 0.97	1.81	1.57; 2.09	1.49	1.37; 1.62
Abnormal platelet count	0.99	0.87; 1.12	1.42	1.21; 1.66	1.21	1.10; 1.33
**Patients with cancer**						
SMOKE						
No	1.0	Ref	1.0	ref	1.0	Ref
Yes	1.25	1.03; 1.53	1.27	0.98; 1.64	1.34	1.22; 1.47
Sex						
Male	1.0	Ref	1.0	ref	1.0	Ref
Female	0.94	0.82; 1.08	1.06	0.89; 1.26	0.86	0.80; 0.91
Therapy duration (months)	0.91	0.90; 0.92	0.88	0.86; 0.90	0.96	0.95; 0.96
Age	0.99	0.98; 1.00	1.00	0.99; 1.01	1.00	1.00; 1.01
Symptomatic PE	0.93	0.81; 1.07	1.52	1.27; 1.81	1.26	1.18; 1.35
Surgery	0.51	0.41; 0.65	0.95	0.74; 1.22	0.50	0.44; 0.56
Immobility	0.91	0.76; 1.11	1.31	1.07; 1.61	1.49	1.39; 1.61
Estrogen intake	0.97	0.73; 1.27	1.09	0.77; 1.54	0.58	0.49; 0.68
Chronic lung disease	1.09	0.89; 1.35	1.22	0.96; 1.54	1.03	0.94; 1.12
Chronic heart failure	1.07	0.79; 1.45	0.90	0.64; 1.26	1.08	0.96; 1.23
Arterial hypertension	1.06	0.91; 1.24	1.05	0.87; 1.27	0.84	0.79; 0.91
Diabetes	1.11	0.92; 1.34	0.97	0.77; 1.22	1.15	1.06; 1.24
CrCL levels						
>60 mL min	1.0	Ref	1.0	ref	1.0	Ref
30–60 mL min	1.03	0.83; 1.23	1.15	0.93; 1.42	1.19	1.10; 1.29
<30 mL min	0.87	0.61; 1.23	1.45	1.04; 2.02	1.61	1.42; 1.82
Anaemia	0.92	0.80; 1.06	1.64	1.35; 1.99	1.55	1.45; 1.66
Abnormal platelet count	1.15	0.99; 1.34	1.28	1.06; 1.54	1.35	1.26; 1.44
	**Patients with EP (with or without DVT)**					
	**VTE Recurrence**		**Major Bleeding**		**Death**	
	**HR**	**95% CI**	**HR**	**95% CI**	**HR**	**95% CI**
SMOKE						
No	1.0	Ref	1.0	ref	1.0	Ref
Yes	1.09	0.93; 1.29	1.28	1.03; 1.60	1.0	1.21; 1.50
Sex						
Male	1.0	Ref	1.0	ref	1.0	Ref
Female	0.96	0.85; 1.07	1.16	1.01; 1.34	0.88	0.82; 0.94
Therapy duration (months)	0.92	0.91; 0.93	0.92	0.91; 0.93	0.96	0.96; 0.97
Age	1.00	1.00; 1.01	1.02	1.01; 1.03	1.03	1.02; 1.03
Symptomatic PE						
Surgery	0.46	0.37; 0.56	1.01	0.82; 1.24	0.59	0.52; 0.67
Immobility	0.82	0.71; 0.94	1.11	0.95; 1.30	1.59	1.49; 1.71
Estrogen intake	0.47	0.35; 0.62	0.92	0.66; 1.28	0.52	0.44; 0.63
Cancer	1.77	1.56; 2.00	1.71	1.48; 1.98	4.68	4.37; 5.01
Chronic lung disease	1.02	0.87; 1.19	1.14	0.96; 1.35	1.17	1.08; 1.27
Chronic heart failure	1.10	0.89; 1.35	1.01	0.82; 1.24	1.48	1.35; 1.62
Arterial hypertension	1.05	0.93; 1.19	1.08	0.93; 1.26	0.78	0.73; 0.84
Diabetes	0.93	0.79; 1.09	0.94	0.79; 1.12	1.10	1.02; 1.19
CrCL levels						
>60 mL min	1.0	Ref	1.0	ref	1.0	Ref
30–60 mL min	1.06	0.92; 1.22	1.28	1.08; 1.51	1.25	1.15; 1.35
<30 mL min	1.09	0.85; 1.40	1.73	1.37; 2.20	2.03	1.82; 2.26
Anaemia	0.89	0.79; 1.01	1.67	1.45; 1.93	1.54	1.44; 1.64
Abnormal platelet count	1.04	0.91; 1.19	1.36	1.17; 1.58	1.36	1.27; 1.46
	**Patients with DVT (no EP)**					
	**VTE Recurrence**		**Major Bleeding**		**Death**	
	**HR**	**95% CI**	**HR**	**95% CI**	**HR**	**95% CI**
SMOKE						
No	1.0	Ref	1.0	ref	1.0	Ref
Yes	1.19	1.02; 1.39	0.96	0.70; 1.31	1.14	1.00; 1.31
Sex						
Male	1.0	Ref	1.0	ref	1.0	Ref
Female	0.84	0.75; 0.95	1.06	0.88; 1.27	0.96	0.88; 1.04
Therapy duration (months)	0.94	0.93; 0.95	0.93	0.91; 0.94	0.87	0.86; 0.88
Age	1.00	0.99; 1.00	1.01	1.00; 1.02	1.02	1.02; 1.03
Symptomatic PE						
Surgery	0.66	0.54; 0.80	0.71	0.52; 0.97	0.51	0.44; 0.59
Immobility	0.98	0.86; 1.12	1.39	1.15; 1.69	1.80	1.66; 1.97
Estrogen intake	0.69	0.52; 0.91	0.65	0.39; 1.07	0.44	0.34; 0.57
Cancer	1.78	1.57; 2.01	1.95	1.61; 2.36	6.32	5.78; 6.92
Chronic lung disease	1.12	0.94; 1.35	1.24	0.94; 1.62	1.25	1.11; 1.41
Chronic heart failure	1.06	0.80; 1.40	1.69	1.26; 2.27	1.45	1.26; 1.66
Arterial hypertension	1.10	0.97; 1.24	1.12	0.91; 1.36	0.83	0.76; 0.90
Diabetes	1.03	0.88; 1.20	0.91	0.72; 1.15	1.35	1.23; 1.49
CrCL levels						
>60 mL min	1.0	Ref	1.0	ref	1.0	Ref
30–60 mL min	1.20	1.03; 1.39	1.39	1.10; 1.75	1.25	1.13; 1.39
<30 mL min	1.02	0.79; 1.33	2.11	1.58; 2.81	1.98	1.74; 2.25
Anaemia	0.94	0.83; 1.06	2.11	1.73; 2.58	1.77	1.62; 1.94
Abnormal platelet count	1.10	0.97; 1.26	1.41	1.16; 1.71	1.36	1.25; 1.49

Legend to Table 4: HR, hazard ratio; CI, confidence intervals; PE, pulmonary embolism; CrCl, creatinine clearance; DVT, deep vein thrombosis; Ref., reference.

## Coordinator of the RIETE Registry

Manuel Monreal.

## RIETE Steering Committee Members

Paolo Prandoni, Benjamin Brenner, and Dominique Farge-Bancel.

## RIETE National Coordinators

Raquel Barba (Spain), Pierpaolo Di Micco (Italy), Laurent Bertoletti (France), Sebastian Schellong (Germany), Inna Tzoran (Israel), Abilio Reis (Portugal), Marijan Bosevski (R. Macedonia), Henri Bounameaux (Switzerland), Radovan Malý (Czech Republic), Peter Verhamme (Belgium), Joseph A. Caprini (USA), and Hanh My Bui (Vietnam).

## RIETE Registry Coordinating Center

S & H Medical Science Service.

## Appendix A

### Members of the RIETE Group

SPAIN: Adarraga MD, Agud M, Aibar J, Aibar MA, Alfonso J, Alonso-Carrillo J, Amado C, Arcelus JI, Baeza C, Ballaz A, Barba R, Barbagelata C, Barrón M, Barrón-Andrés B, Blanco-Molina A, Botella E, Camon AM, Casado I, Castro J, Criado J, de Ancos C, de Miguel J, del Toro J, Demelo-Rodríguez P, Díaz-Pedroche C, Díaz-Peromingo JA, Díaz-Simón R, Díez-Sierra J, Domínguez IM, Encabo M, Escribano JC, Falgá C, Farfán AI, Fernández-Capitán C, Fernández-Reyes JL, Fernández de Roitegui K, Fidalgo MA, Flores K, Font C, Font L, Francisco I, Furest I, Gabara C, Galeano-Valle F, García MA, García-Bragado F, García-Hernáez R, García-Raso A, Gavín-Sebastián O, Gil-Díaz A, Gómez-Cuervo C, González-Martínez J, Grau E, Giménez-Suau M, Guirado L, Gutiérrez J, Hernández-Blasco L, Hernando E, Jara-Palomares L, Jaras MJ, Jiménez D, Joya MD, Jou I, Lalueza A, Lecumberri R, Lima J, Llamas P, Lobo JL, López-Jiménez L, López-Miguel P, López-Núñez JJ, López-Reyes R, López-Sáez JB, Lorente MA, Lorenzo A, Loring M, Madridano O, Maestre A, Marchena PJ, Martín del Pozo M, Martín-Martos F, Martínez-García MA, Mella C, Mellado M, Mercado MI, Moisés J, Monreal M, Morales MV, Muñoz-Blanco A, Muñoz-Guglielmetti D, Muñoz-Rivas N, Nieto JA, Núñez-Ares A, Núñez-Fernández MJ, Obispo B, Olivares MC, Orcastegui JL, Ortega-Recio MD, Osorio J, Otalora S, Otero R, Parra P, Parra V, Pedrajas JM, Pellejero G, Pesántez D, Porras JA, Portillo J, Redondo-Martínez I, Riera-Mestre A, Rivas A, Rivera F, Rodríguez-Cobo A, Rodríguez-Matute C, Rogado J, Rosa V, Rubio CM, Ruiz-Artacho P, Ruiz-Giménez N, Ruiz-Ruiz J, Ruiz-Sada P, Sahuquillo JC, Salgueiro G, Sampériz A, Sánchez-Muñoz-Torrero JF, Sancho T, Sigüenza P, Soler S, Suárez S, Suriñach JM, Torres MI, Tolosa C, Trujillo-Santos J, Uresandi F, Valle R, Vela JR, Vidal G, Villares P, and Zamora C. ARGENTINA: Gutiérrez P, and Vázquez FJ. BELGIUM: Vanassche T, Vandenbriele C, and Verhamme P. CZECH REPUBLIC: Hirmerova J and Malý R. ECUADOR: Salgado E. FRANCE: Benzidia I, Bertoletti L, Bura-Riviere A, Crichi B, Debourdeau P, Espitia O, Farge-Bancel D, Helfer H, Mahé I, Moustafa F, and Poenou G. GERMANY: Schellong S. ISRAEL: Braester A, Brenner B, and Tzoran I. ITALY: Bilora F, Brandolin B, Ciammaichella M, Colaizzo D, Dentali F, Di Micco P, Giorgi-Pierfranceschi M, Grandone E, Mastroiacovo D, Maida R, Mumoli N, Pace F, Pesavento R, Pomero F, Prandoni P, Quintavalla R, Rocci A, Siniscalchi C, Tufano A, Visonà A, and Zalunardo B. LATVIA: Gibietis V, Kigitovica D, and Skride A. PORTUGAL: Ferreira M, Fonseca S, Martins F, and Meireles J. REPUBLIC OF MACEDONIA: Bosevski M. SWITZERLAND: Bounameaux H, and Mazzolai L. USA: Caprini JA, Tafur AJ, Weinberg I, and Wilkins H. VIETNAM: Bui HM.

## References

[1] GBD 2016 Mortality and Causes of Death Collaborators (2017). Global, regional, and national age-sex specific mortality for 264 causes of death, 1980–2016: A systematic analysis for the Global Burden of Disease Study 2016. Lancet.

[2] Banks E., Joshy G., Korda R.J., Stavreski B., Soga K., Egger S., Day C., Clarke N.E., Lewington S., Lopez A.D. (2019). Tobacco smoking and risk of 36 cardiovascular disease subtypes: Fatal and non-fatal outcomes in a large prospective Australian study. BMC Med..

[3] Piepoli M.F., Hoes A.W., Agewall S., Albus C., Brotons C., Catapano. A.L., Cooney M.T., Corrà U., Cosyns B., Deaton C. (2016). 2016 European Guidelines on cardiovascular disease prevention in clinical practice: The Sixth Joint Task Force of the European Society of Cardiology and Other Societies on Cardiovascular Disease Prevention in Clinical Practice (constituted by representatives of 10 societies and by invited experts) Developed with the special contribution of the European Association for Cardiovascular Prevention & Rehabilitation (EACPR). Eur. Heart J..

[4] Mahmoodi B.K., Cushman M., Anne Næss I., Allison M.A., Bos W.J., Brækkan S.K., Cannegieter S.C., Gansevoort R.T., Gona P.N., Hammerstrøm J. (2017). Association of Traditional Cardiovascular Risk Factors With Venous Thromboembolism: An Individual Participant Data Meta-Analysis of Prospective Studies. Circulation.

[5] Ezzati M., Henley S.J., Thun M.J., Lopez A.D. (2005). Role of smoking in global and regional cardiovascular mortality. Circulation.

[6] Ageno W., Becattini C., Brighton T., Selby R., Kamphuisen P.W. (2008). Cardiovascular risk factors and venous thromboembolism: A meta-analysis. Circulation.

[7] Cheng Y.J., Liu Z.H., Yao F.J., Zeng W.T., Zheng D.D., Dong Y.G., Wu S.H. (2013). Current and former smoking and risk for venous thromboembolism: A systematic review and meta-analysis. PLoS Med..

[8] Bikdeli B., Jiménez D., Hawkins M., Ortíz S., Prandoni P., Brenner B., Decousus H., Masoudi F.A., Trujillo-Santos J., Krumholz H.M. (2018). Rationale, design and methodology of the computerized registry of patients with venous thromboembolism (RIETE). Thromb. Haemost..

[9] Carruzzo P., Méan M., Limacher A., Aujesky D., Cornuz J., Clair C. (2016). Association between smoking and recurrence of venous thromboembolism and bleeding in elderly patients with past acute venous thromboembolism. Thromb. Res..

[10] Linnemann B., Zgouras D., Schindewolf M., Schwonberg J., Jarosch-Preusche M., Lindhoff-Last E. (2008). Impact of sex and traditional cardiovascular risk factors on the risk of recurrent venous thromboembolism: Results from the German MAISTHRO Registry. Blood Coagul Fibrinolysis.

[11] Enga K.F., Braekkan S.K., Hansen-Krone I.J., le Cessie S., Rosendaal F.R., Hansen J.B. (2012). Cigarette smoking and the risk of venous thromboembolism: The Tromsø Study. J. Thromb. Haemost..

[12] Wattanakit K., Lutsey P.L., Bell E.J., Gornik H., Cushman M., Heckbert S.R., Rosamond W.D., Folsom A.R. (2012). Association between cardiovascular disease risk factors and occurrence of venous thromboembolism. A time-dependent analysis. Thromb. Haemost..

[13] Paulsen B., Gran O.V., Severinsen M.T., Hammerstrøm J., Kristensen S.R., Cannegieter S.C., Skille H., Tjønneland A., Rosendaal F.R., Overvad K. (2021). Association of smoking and cancer with the risk of venous thromboembolism: The Scandinavian Thrombosis and Cancer cohort. Sci. Rep..

[14] Critchley J.A., Capewell S. (2003). Mortality risk reduction associated with smoking cessation in patients with coronary heart disease: A systematic review. JAMA.

[15] Lightwood J.M., Glantz S.A. (1997). Short-term economic and health benefits of smoking cessation: Myocardial infarction and stroke. Circulation.

[16] Ambrose J.A., Barua R.S. (2004). The pathophysiology of cigarette smoking and cardiovascular disease: An update. J. Am. Coll. Cardiol..

[17] Napoli C., Ignarro L.J. (2001). Nitric oxide and atherosclerosis. Nitric. Oxide.

[18] Loscalzo J. (2001). Nitric oxide insufficiency, platelet activation, and arterial thrombosis. Circ. Res..

[19] Blache D. (1995). Involvement of hydrogen and lipid peroxides in acute tobacco smoking-induced platelet hyperactivity. Am. J. Physiol..

[20] Hung J., Lam J.Y., Lacoste L., Letchacovski G. (1995). Cigarette smoking acutely increases platelet thrombus formation in patients with coronary artery disease taking aspirin. Circulation.

[21] Benowitz N.L., Fitzgerald G.A., Wilson M., Zhang Q. (1993). Nicotine effects on eicosanoid formation and hemostatic function: Comparison of transdermal nicotine and cigarette smoking. J. Am. Coll. Cardiol..

[22] Barua R.S., Ambrose J.A. (2013). Mechanisms of coronary thrombosis in cigarette smoke exposure. Arterioscler. Thromb. Vasc. Biol..

[23] Holst A.G., Jensen G., Prescott E. (2010). Risk factors for venous thromboembolism: Results from the Copenhagen City Heart Study. Circulation.

[24] Gandini S., Botteri E., Iodice S., Boniol M., Lowenfels A.B., Maisonneuve P., Boyle P. (2008). Tobacco smoking and cancer: A meta-analysis. Int. J. Cancer.

[25] Colpani V., Baena C.P., Jaspers L., van Dijk G.M., Farajzadegan Z., Dhana K., Tielemans M.J., Voortman T., Freak-Poli R., Veloso G.G.V. (2018). Lifestyle factors, cardiovascular disease and all-cause mortality in middle-aged and elderly women: A systematic review and meta-analysis. Eur. J. Epidemiol..

[26] Studts J.L., Ghate S.R., Gill J.L., Studts C.R., Barnes C.N., LaJoie A.S., Andrykowski M.A., LaRocca R.V. (2006). Validity of self-reported smoking status among participants in a lung cancer screening trial. Cancer Epidemiol. Biomark. Prev..

